# Supplementary material for: Are carbon emissions trading and green financial instruments synergistic? -Comprehensive quantitative research based on content analysis
Source: PLoS One. 2024 Mar 7;19(3):e0298601. doi: 10.1371/journal.pone.0298601 (PMC10919601; doi:10.1371/journal.pone.0298601)
Supplement: S1 Data — (ZIP) [file pone.0298601.s001.zip › Data/地级市Z_F工作报告.zip!/╣π╢1⁄2/╗▌╓▌╩╨/└┤╫╘╣1⁄2╓┌║┼í╛┬φ┐╦╩2╛▌═°í┐_2014.docx]

各位代表：

　　现在，我代表市人民政府向大会报告工作，请予审议，并请政协委员和其他列席人员提出意见。

　　2013年工作回顾

　　2013年是惠州发展提速之年、改革深化之年、民生改善之年。一年来，在省委、省政府和市委的正确领导下，在市人大和市政协的监督支持下，市政府紧紧围绕 “尽快进入珠三角第二梯队”这一奋斗目标，突出“稳中求进、好中求快、率先跨越”，大力实施“六大计划”，积极应对复杂多变的经济形势，全力克服特大自然灾害的不利影响，奋发进取，扎实工作，完成了市十一届人大三次会议确定的各项目标任务，争先进位取得实质性进展。

　　（一）经济综合实力进一步增强。全市实现生产总值2678.4亿元，比上年（下同）增长13.6%；地方公共财政预算收入250.1亿元，增长24.5%，经济总量、财政实力同步从全省第六位迈进第五位，增幅均居全省第三位、珠三角首位。规模以上工业增加值1374.6亿元，增长17.7%。固定资产投资1401.3亿元，增长18.6%，其中重点建设项目投资453.2亿元，超额完成年度计划。社会消费品零售总额857.9亿元，增长13.5%。外贸进出口573.9亿美元，增长16%；其中出口333.2亿美元，增长14.1%。实际利用外商直接投资18.3亿美元，增长6.2%。大力实施促进实体经济发展的新 “惠28条”和促进民营经济发展的“惠42条”，深入开展企业服务月活动，开通企业服务集市网上平台，全力帮扶企业拓展市场、加快发展。成功举办惠州产品呼和浩特展销会、莫斯科展览会，签订销售合同164亿元。新增规模以上工业企业360家、民营企业245家，129家企业增资扩产。惠州三星电子成为首个产值超千亿元企业。全市工业用电量176.9亿千瓦时，增长10.5%。落实《珠三角规划纲要》“四年大发展”考核获评优秀等级，入选“中国最具海外影响力城市”[1]。

　　（二）产业转型升级进一步加快。先进制造业增加值920亿元，增长20%，占规模以上工业增加值66.9%。电子信息产业增加值560.2亿元，增长29.3%。石油化工产业增加值314.1亿元，增长9.2%。仲恺高新区成为国家首批创新型产业集群建设试点、新型工业化移动智能终端产业示范基地、LED产业基地。中海油惠炼二期、信利AMOLED等项目动工建设，中海油乐金ABS、比亚迪锂电池、TCL移动互联网智能终端、元晖LED照明等项目建成投产。新增省级农业龙头企业10家、名优农产品9个，16个种植基地入选省级“菜篮子”基地。成功举办广东省道教文化节暨第七届国际旅游节、第二届“云博会”、第三届“农博会”。全年接待旅游人数3551.5万人次，旅游总收入212.7亿元，分别增长12.6%和15.5%。罗浮山成功创建国家5A级景区，叶挺将军纪念园获评国家4A级景区。惠州港年吞吐量突破8000万吨。组建惠东农商行、博罗农商行，新引进金融机构13家，天津股权交易所惠州运营中心、前海股权交易中心惠州办事处挂牌营业。全市金融机构本外币存贷款余额分别为3138.8亿元和2036.9亿元，分别比年初增长16.4%和17.4%，存贷比达64.9%。三次产业比例调整为5.1∶57.9∶37，第三产业占比提高0.5个百分点。全市产业转型升级成效明显，走出了一条“靠引进大型企业、引进先进生产力”的转型升级之路。

　　（三）区域协调发展进一步加强。环大亚湾新区上升为全省发展战略。“县区赶超计划”深入实施，各县区动竣工项目合计达到735宗，投资总额超过3700亿元。惠城区获评全国社会管理综合治理先进集体，人均GDP超过全国平均水平。惠阳区获评全国法治县区创建活动先进单位，现代服务业和先进制造业同步发展，伯恩光学产值超百亿元。惠东县滨海旅游业快速发展，稔平半岛五星级酒店群初具规模，惠东产业转移园实现规模以上工业总产值150.5亿元，增长84.2%。博罗县通过省文明县城验收，实现人均GDP超过全国平均水平，跻身广东县域经济综合发展力前三甲。龙门县蝉联“中国温泉之乡”，连续三年获评“最受欢迎省内游目的地”。大亚湾开发区石化产业规模不断壮大，新引进石化中下游项目15宗，总投资152亿元，东风本田一体化项目顺利落地。仲恺高新区科技创业服务中心等孵化器功能持续增强，GDP、地方公共财政预算收入等主要经济指标增幅均居全市第一。

　　（四）城乡基础设施进一步完善。完成基础设施投资266.5亿元，增长27%。厦深铁路惠州段建成通车，莞惠城轨惠州段建设加快。开展高速公路建设 “百日攻坚战”，7条在建高速公路建设进展良好。国际集装箱码头一期泊位正式启用，首条惠州至上海跨省内贸集装箱航线开通。改造完成惠州大道东段、机场路一期和三环路，打通市区17条连接路，新建4座人行天桥和文昌地下通道。新湖公园、文星公园建成开园。新开通公交线路22条，被确定为全省首批创建公共交通示范城市。完成县道改造53.1公里、通自然村公路硬底化800公里。建成潼湖水厂主体工程和16宗农村饮水安全工程，市区供水一体化取得阶段性成果。惠州大堤南堤、潼湖围达标加固等水利工程建设顺利推进。建成高标准基本农田25万亩、粮食高产示范片102个。500千伏和220千伏输变电工程建设快速推进，新增主变容量98万千伏安。

　　（五）重点领域改革进一步深化。出台第五轮行政审批制度改革第二批调整目录，取消、下放行政许可事项51项，开展行政审批标准化建设试点工作，向社会公示政府权责清单。在市级层面推行投资项目审批制度改革，统一受理申报，优化审批流程，实施“并联办理”、“联合审查”，审批提速80%。全面推广商事登记制度改革，实行注册资本“零首期”和“认缴制”，工商登记注册的内资企业增长33.8%。网上办事大厅服务功能全面提升，实现“横向覆盖部门，纵向延伸县区”，6494项服务事项100%进驻，可在线办理6244项，超出省定标准，累计网上办事突破1000万宗。公共资源交易改革向基层延伸，全部中心镇建成公共资源交易所，市、县区、镇办三级交易平台成交总额300亿元，增收节支超过20亿元。社会信用体系、市场监管体系建设扎实推进，考核排名全省第二。食品药品监管体制改革、卫生和计生机构改革基本完成，医药卫生体制改革、事业单位分类改革进展顺利。荣获“中国改革十大最具投资潜力城市”称号。

　　（六）生态环境质量进一步提升。淡水河、潼湖流域污染整治继续深化，在省人大组织的第三方评估考核中排名第二。实施青年河综合整治。扎实推进林业四大重点工程建设，建成县级以上森林公园8个、森林家园100个、森林绣花工程7个、千村家园绿化示范村10个，森林覆盖率提高到61.28%，城市人均公园绿地面积提高到16.8平方米。整治违法用地违法建设工作扎实推进。建成污水处理厂21座、截污管网176公里，城镇污水处理率达到95.1%。加大淘汰营运黄标车和老旧车力度，机动车污染减排名列全省第一。单位GDP能耗完成省下达任务。完成市垃圾焚烧发电厂升级改造，惠城中心区推行垃圾分类处理。“美丽乡村·清洁先行”活动深入开展，全面完成城乡生活垃圾处理设施年度建设任务。建成国家级生态镇8个、省级生态镇25个。连续六年获全省环保责任考核优秀等级，入选“中国十佳空气质量城市”、“中国十佳宜居城市”。绿色和平组织发布的2013年中国74个城市PM2.5年均浓度排名，我市排在污染最轻的城市第7位。

　　（七）民生保障力度进一步加大。全市公共财政民生支出224.8亿元，增长22.6%，占公共财政预算支出比重从上年的66.9%提高到68.5%。十件民生实事66个项目全部完成。基本公共服务均等化综合改革14个专题、219个项目全部完成，提前基本实现国家基本公共服务体系“十二五”规划目标。新增城镇就业人员6.56万人，转移农村富余劳动力1.1万人，城镇登记失业率控制在2.35%。城镇居民人均可支配收入33300元，农村居民人均纯收入14029元，分别增长11.1%和13%。城乡居民社会养老保险基础养老金从每人每月70元提高到90元，企业退休人员人均养老金从每人每月1616元提高到1776元。城乡低保标准统一提高到每人每月430元。建成市社会福利服务中心一期主体工程。城乡居民基本医疗保险财政补助标准从每人每年252元提高到300元，高于省定标准20元。门诊特定病种项目从21种增加到26种。实行大病二次补偿，报销比例从50%提高到95%。中大惠亚医院建成开业；完成村卫生站标准化建设；平价医疗服务范围不断扩大。新建平价商店61家，向低收入群体发放价格补贴600万元。建设保障性住房3385套，超额完成省下达任务。完成农村困难户住房改造4348户、水库移民住房改造428户、华侨农场危房改造1000户。开展新一轮扶贫开发，投入帮扶资金1.3亿元。成功抗击“8·16”洪灾和强台风“天兔”，救灾复产、重建家园全面完成，受灾全倒户全部搬进新居。我市被确定为省社会创新实验基地，“特色之家[2]”成为省社会创新试点项目。信访维稳、安全生产、食品药品监管持续加强。社会治安综合治理深入开展，全警上路“大巡防”全面铺开，“雷霆扫毒”行动取得重大成效，制毒贩毒、“两抢一盗”等违法犯罪案件明显下降，群众安全感明显提升，入选“中国最安全城市”，再次荣获“全国社会管理综合治理优秀市”，连续第四次获评“中国最具幸福感城市”。

　　（八）社会各项事业进一步发展。科技创新成效显著。全市研发经费支出占GDP比重提高到2.1%，新增国家级工业设计中心1家、省级专业镇2个、省级工程技术研发中心15家，获新认定高新技术企业37家、省级创新型企业4家，第五次获评全国科技进步考核工作先进市，入选全国智慧城市试点市、信息消费试点市。教育事业加快发展。四个区通过省推进教育现代化先进区督导验收。新增优质幼儿园91所，新建和改扩建中小学校18所，新增公办学位1万个。成为推进义务教育均衡发展国家教育体制改革示范市。惠州商务职业学院完成省高等学校设置评议。2.3万名困难中小学生吃上免费营养午餐，异地务工人员随迁子女享受电子教育券补助。文体事业更加繁荣。全国城市文明程度指数测评名列全省地级市第一，文化惠民卡成功竞得创建国家第二批公共文化服务体系示范项目。建成报业传媒集团全媒体产业基地，完成广播电视户户通工程。银岗古窑场纳入全省八大考古遗址公园建设计划。成功举办第二届国际龙舟邀请赛、第十三届世界象棋锦标赛、第四届市运会等重大赛事。人口计生工作取得新突破。双拥共建、国防教育、人民防空、民兵预备役等工作迈出新步伐。妇女儿童、老年人、残疾人、红十字等事业取得新发展。外事、侨务、港澳、台务等工作取得新成效。民族、宗教、统计、方志、档案、移民、仲裁、气象、三防、消防、应急、防震、口岸、打私等工作取得新进步。

　　与此同时，政府自身建设进一步加强，法治型、服务型、责任型、效能型、和谐型、廉洁型政府建设扎实推进。认真执行市人大及其常委会决议，坚持向市人大常委会报告工作，及时向市政协常委会通报政务，自觉接受人大的法律监督和政协的民主监督。办理人大代表议案12件、建议171件，政协提案194件、委员意见建议209条，办复率100%。新生儿疾病免费筛查的议案圆满结案。积极开展审计、监察工作，认真落实政务公开、专家咨询、社会公示和社会听证等制度，广泛开展“万众评公务”、“党政领导信箱”、“行风热线”、“惠民在线”等活动。深入推进法治建设，“法制副主任”制度实现全覆盖，获评全国法治城市创建活动先进单位。严格执行中央八项规定，认真落实国务院“约法三章”，坚决厉行节约，市本级“三公”经费下降9.6%，政府廉政建设进一步加强。开展“服务型机关建设年”活动，政府行政效率和服务水平不断提升，政府公共服务总体满意度蝉联全省第一。

　　各位代表，过去一年的成绩来之不易，饱含着省委、省政府的深切关怀，凝聚了全市人民的智慧汗水，离不开海内外各界人士的关心支持。在此，我代表市人民政府，向辛勤工作在各行各业的全市干部群众，向大力支持政府工作的人大代表、政协委员、各民主党派、工商联和各界人士，向关心帮助地方发展的驻惠部队、武警官兵、驻惠单位，向积极参与惠州建设的港澳台同胞、海外侨胞、国际友人，表示衷心的感谢并致以崇高的敬意！

　　我们同时清醒地认识到，我市经济社会发展和政府工作仍存在一些困难和问题。主要表现在：一是经济实力还不够雄厚，民营经济发展不够充分，现代服务业和战略性新兴产业规模不大，产业结构调整优化有待加强；二是科技人才仍然不足，自主创新能力有待增强；三是节能减排压力较大，推进集约节约发展有待加强；四是区域城乡发展不平衡，城镇化水平有待提高；五是制约发展的思想观念、体制机制等因素仍然存在，改革有待全面深化；六是城市治理有待进一步完善，治理能力有待提高；七是食品安全、安全生产等工作有待进一步加强；八是“四风”问题还不同程度存在，政府职能转变、行政效能提升还需作出更大努力。对于这些问题，我们一定高度重视，并采取有力措施，切实加以解决。

　　2014年工作安排

　　2014年是深入贯彻落实党的十八届三中全会精神、全面深化改革的第一年，也是我市推动跨越发展、尽快进入珠三角第二梯队的关键一年。当前，我市既面临着做大经济总量与提升发展质量的双重任务，又肩负着优化生态环境与改善民生质量的双重责任。尽管宏观经济形势仍有许多不稳定、不确定因素，前进的道路上还有很多困难和挑战，但总的来说我市处于大可作为的重要战略机遇期，具备跨越发展的坚实基础和良好条件。党的十八届三中全会作出了全面深化改革的战略部署，为我市改革发展提供了新的重大机遇；省委、省政府推出一系列改革举措，实施扩大内需、创新驱动、协调发展等战略措施，为我们加快转型升级提供了有力支持；历届市委、市政府带领全市人民努力拼搏，打下了良好的经济社会发展基础；市委立足惠州发展新要求，吹响了尽快进入珠三角第二梯队的号角，成为激励全市人民奋发进取的强大动力；环大亚湾新区上升为省的发展战略，惠州正式迈入“高铁时代”。所有这些，都必将激励惠州人民更加奋发有为。只要我们坚定信心，真抓实干，奋力拼搏，就一定能够在新的起点上推动新发展、开创新局面、实现新跨越。

　　2014年政府工作的总体思路是：全面贯彻党的十八大、十八届三中全会、中央经济工作会议和省委十一届三次全会精神，按照市第十次党代会第三次会议、市委十届五次全会的工作部署，坚持“稳中求进、好中求快、改革创新、率先跨越”，围绕三大核心任务，大力实施“六大计划”和“六个工作方案[3]”，全面深化改革，加快转型升级，促进优质增长，办好民生实事，推进我市争先进位、跨越发展，打造惠州经济升级版，为实现尽快进入珠三角第二梯队奋斗目标奠定坚实基础。

　　经济社会发展的主要预期目标是：生产总值增长11%；人均生产总值增长10%；固定资产投资增长18.5%；社会消费品零售总额增长13.5%；外贸出口增长5%；地方公共财政预算收入增长13%；城镇居民人均可支配收入增长11%；农民人均纯收入增长12%；居民消费价格指数控制在3.5%以内；服务业增加值占生产总值的37%左右；城镇登记失业率控制在2.7%以内；户籍人口自然增长率完成省下达任务；全社会研发经费支出占生产总值比重2.2%；节能减排各项指标完成省下达任务。

　　今年政府工作必须坚持以改革增动力、以创新促转型、以发展惠民生，大力推进八项重点工作：

　　（一）努力扩大有效需求，确保经济持续快速发展。

　　坚持发展第一要务，扭住经济建设中心不动摇，充分发挥消费的基础作用、投资的关键作用和出口的支撑作用，努力形成消费、投资、出口协调拉动经济增长的格局。

　　扩大消费需求。深入推进惠货全球行、全国行、网上行，举办惠州产品南非展览会、济南展销会。深入实施电子商务发展规划，加强与国内外大型电商平台合作，启动建设电子商务产业园。加快4G网络建设和业务开展，扩大信息消费。推进万村千乡市场工程建设和农超对接。鼓励支持企业在国内大中城市开设品牌产品展销中心，组织开展各种促销活动，努力扩大内销市场。

　　促进投资增长。全力推进一批关系长远、带动全局的重大基础设施、重大产业项目和重大民生项目建设，不断增强经济发展后劲。全年安排重点项目189项，总投资3518亿元，年度计划投资459.8亿元。其中新开工项目71项，续建项目94项，投产项目24项。做好重点项目谋划储备和前期工作，促进重点项目建设有序接替、梯次发展。

　　强化招商选资。举办第三届云博会和第十三届香港珠三角工商界合作交流会，利用各种展会平台，积极引进世界500强企业、跨国集团、大型央企省企、国内100强企业和成长型、技术型新兴产业项目，实现利用外资数量和质量同步提升。做好对现有企业的服务，促进企业增资扩产、做大做强。

　　优化出口结构。坚持重点企业联系制度，全力帮扶出口大户扩大外贸出口。大力支持自主知识产权、自主品牌、自主营销商品出口，扩大机电和高新技术产品出口。组织企业参加广交会、高交会等重大经贸活动，巩固传统市场，拓展新兴市场。发展出口信用保险服务，提高企业抗风险能力。加强口岸建设，进一步优化通关环境。

　　壮大实体经济。大力实施“企业成长计划”，新培育年主营业务收入超千亿元、超百亿元企业各1家。完善促进实体经济发展政策措施，深入开展企业服务月活动，切实帮助企业解决生产经营中遇到的困难。认真落实扶持民营经济发展政策措施，全力营造公平竞争、平等准入的市场环境，鼓励民营资本参与基础设施建设、新兴产业发展。支持民营企业科技创新、自营出口，推动民营经济转型升级。

　　（二）全面深化各项改革，增强发展动力和活力。

　　把深化改革贯穿于经济社会发展各个领域各个环节，加大改革攻坚力度，全面落实今年50项改革重点任务，以新一轮大改革推动我市新一轮大发展。

　　深化经济体制改革。完善工商注册制度改革，全面实行注册资本 “零首期”、“认缴制”。落实“非禁即入”投资政策，实行投资管理负面清单管理制度。深入推进市场监管体系建设，强化企业信用监管。进一步推进国有资产授权经营体制改革，支持有条件的国有企业改组为国有资本投资公司。完善公共资源交易制度，扩大公共资源交易范围。

　　深化行政体制改革。加快转变政府职能，深化行政审批制度改革。组建市政府决策咨询委员会，提高科学决策水平。推进行政审批标准化建设，完善政府权责清单制度。完成新一轮市、县（区）政府机构改革。理顺城管执法体制，提高执法服务水平。完善网上办事大厅功能，出台《惠州市网上办事管理规范》，推动网上办事大厅向基层延伸。完善投资项目审批窗口服务，加快推行投资审批网上办理。推进财政管理体制改革，完善预算编制和国库集中收付制度，建立全口径的预算编报体系，实施全面规范的预算公开。

　　深化城乡综合改革。编制实施主体功能区规划、城镇化发展规划。稳妥推进户籍制度改革，尽快建立积分入户制度，完善新型城镇化发展机制。推行不动产确权登记。完成农村集体建设用地确权登记发证工作，探索开展集体经营性建设用地使用权流转。启动农村土地承包经营权确权登记颁证试点。建立健全农村集体经济组织，在有条件的村推行农村集体经济股份制改革。扩大政策性农业保险范围，健全农业农村巨灾风险事故救助机制。

　　深化文化体制改革。继续推进国有经营性文化单位改革，加快公司制、股份制改造，创新国有文化资产监督、考核。深化广电、报业传媒集团内部改革，完善法人治理结构和现代企业管理制度。深化公益性文化事业单位绩效工资改革、聘用制改革和岗位责任制改革。明确不同文化事业单位功能定位，完善事业单位法人治理结构，启动组建公共图书馆、博物馆、文化馆理事会工作。加强村级文化协管员队伍建设，创新镇街、村居基层文化工作模式和公共文化服务。

　　深化社会治理改革。实施社会建设“十大行动计划”，不断提升社会治理现代化水平。坚持源头防范治理，完善社会矛盾纠纷化解、社会稳定风险评估机制。强化社区建设，实现政府行政管理与基层群众自治有效衔接。加强互联网管理工作，建立网络舆情信息报送平台，提升舆情预警能力。加快培育和规范管理社会组织，力争全市社会组织达到2700个以上。抓紧制订政府向社会组织转移职能事项清单，积极扩大政府向社会组织购买服务。完善“法制副主任”运行机制和保障机制，建立推广法律顾问制度，深入开展法治示范创建活动。积极申报具有地方立法权的较大的市。

　　（三）大力推进平台建设，打造加快发展的强力引擎。

　　以环大亚湾新区上升为省发展战略为契机，大力实施“平台构建计划”，培育经济发展的核心增长极。

　　全力推进环大亚湾新区建设。抓紧完成环大亚湾新区空间发展、产业发展、基础设施、生态低碳等专项规划，完善新区建设规划体系。加快建立精简高效、运行科学的新型管理体制，加强统筹协调、资源整合，加快推动28平方公里的起步区建设，做大做强石油化工、电子信息、装备制造、滨海旅游、现代服务业等优势产业，着力构建“石化为基、多元发展、高端为本、创新引领”的产业格局。

　　大力提升仲恺高新区发展水平。抓紧编制总体规划，高标准建设潼湖智慧城。加快建设孵化器集群、留学人员创业园和科技与金融服务中心，努力聚集科研院所、高新技术、科技金融、产业资本和高端人才，推动“惠州制造”向“惠州创造”升级。积极推进液晶产业园、移动互联网产业园、LED产业园、新能源产业园和“慧云”创意产业园建设，努力打造全国一流的“现代创新型、生态城市型”高新区。

　　积极推进产业园区扩能增效。科学规划，优化布局，高起点、大力度推进各地产业园区建设，力争取得实质性进展。重点推进惠南高新产业园、东江高新产业园、惠阳经济技术开发区、惠东产业转移园、龙门产业转移园等园区扩容增效，形成优势互补、各具特色、协调发展的园区发展新格局。

　　（四）着力推动转型升级，加快构建现代产业体系。

　　围绕打造四个超千亿元产业集群，大力实施“产业培育计划”，加快构建更具实力的现代产业体系。

　　发展壮大石油化工产业。大力推进中海油惠炼二期项目建设，积极推动LPG制烯烃项目落地，尽早谋划中海油惠炼三期项目。推进大亚湾精细化工园区建设，发挥“隔墙供应”优势，大力引进石化中下游项目、精细化工项目，延伸石化产业链，进一步壮大石化产业规模。

　　加快发展高端电子信息产业。大力推进信利AMOLED项目、TCL6代线面板等系列项目、粤泰信新型电子扩散板项目、北斗装备制造和应用产业基地、中兴新通讯基地、雅图光电数字视频产业基地建设，大力发展新型显示、新一代通信、数字装备制造等高端电子信息产业，进一步提升电子信息产业整体实力和综合竞争力。

　　积极发展汽车和新能源产业。大力推进东风本田一体化项目、京兰新能源汽车产业项目建设，抓好博罗、惠阳、大亚湾汽车产业园区建设，积极发展汽车整车、汽车关键零部件和新能源汽车等高附加值项目，努力扩大汽车产业规模。抓好LNG电厂扩建、惠州国家石油储备基地等能源续建项目建设，开工建设省天然气管网三期工程惠州段项目，不断做大做强汽车和新能源产业。

　　提升现代服务业发展水平。放大罗浮山成功创建国家5A级景区的效应，大力推进环南昆山旅游产业园建设，抓好西湖创5A工作，完善巽寮湾滨海旅游设施，加快发展现代旅游业。新引进商业银行2家，抓紧组建龙门农商行，创办村镇银行3家，完善助农取款“村村通”工程，启动惠东、龙门农村金融服务站建设。推进金融物流公共服务平台建设，促进金融与物流融合发展。完善多层次资本市场，加快发展现代金融业。推进金泽国际物流园三期、粤东商贸物流园和惠阳安博物流园等项目，抓紧“空港”、“海港”经济区建设，力争惠州港吞吐量突破9000万吨，加快发展现代物流业。落实发展总部经济的各项优惠措施，积极培育一批本土总部企业，引进一批国内外企业总部。培育发展研发设计、商务中介、文化创意、科技信息、法律服务等新兴服务业。

　　着力打造智慧高地。发展物联网、云计算等新一代信息技术，推进信息化与工业化深度融合。加强信息基础设施建设，加快宽带网络发展，进一步完善光纤入户。建设市住房公积金运营与监管系统、数字化城市管理信息系统项目一期。启动 “智慧菜篮子示范工程”试点。积极推动智慧旅游建设，提升旅游信息服务智能化水平。深入开展国家智慧城市试点工作，实施智慧惠州建设总体规划，努力把惠州建设成为国内一流、国际知名的智慧高地。

　　（五）深入实施创新驱动，不断提升科技创新能力。

　　坚持把创新驱动发展作为核心战略，完善创新体系，构建创新平台，促进科技创新成果产业化。

　　完善科技创新体制。全面落实科技创新政策，突出企业技术创新主体地位。切实加大科技投入，推动科技与金融结合，积极创办银行科技支行、科技小额贷款公司、科技融资担保公司，通过创业投资、贷款贴息等方式，扶持企业技术创新。实施知识产权战略，推进知识产权示范市建设。

　　推进联合协同创新。深化与暨南大学、广东工业大学的科技合作，共同建设协同创新中心，加快推进与中科院自动化研究所共建先进制造产业技术研究中心。加强中韩、中乌、中以科技合作，加快推动中韩科技园、中乌国际技术转移中心建设。

　　大力构建创新平台。加快推进石油化工研究院、电子信息研究院、元晖LED产业公共技术、武汉大学惠州研究院、北京化工大学惠州研究院等创新平台建设。扩大异地孵化器布局，加快形成孵化集群。鼓励企业与国内外科研机构开展科技合作，大力扶持企业建设研发中心，力争新增创新平台15家、高新技术企业20家。

　　加强人才队伍建设。大力实施人才“双高”计划，力争引进10个以上科技创新团队、30名以上高水平科技领军人才，培养、引进1万名以上高素质的技能型人才，全市高技能人才总量达12万人，为科技创新提供有力的人才保障和智力支撑。

　　（六）切实推动新型城镇化，促进区域城乡协调发展。

　　坚持以人为本、优化布局、完善功能、协调发展，扎实推进新型城镇化建设。编制实施新型城镇化规划，健全新型城镇化发展体制机制，有序推进农业转移人口市民化，引导小城镇专业化、集约化、特色化发展，推动区域协调发展、城乡统筹发展。

　　进一步完善城乡基础设施。加快推进莞惠城轨惠州段建设。力争惠州机场年底恢复民航。继续抓好7条在建高速公路建设。加快完善市区三新北路东段、江北新旺路等10条连接路。启动建设市中心客运枢纽站，推进四环南路、仲恺大道、惠新大道、金恺大道、园洲龙江东江大桥和省道119线、120线、355线、358线惠州段等道路建设。建成荃湾港区国际集装箱码头二号泊位、中海油物流基地码头二期工程。抓紧推进500千伏海东线、220千伏鹿江线等输变电工程建设。

　　进一步提升城市建设管理水平。高起点、高标准规划建设城市中轴线，大力推进南部新城、东部新城开发建设，整治美化市区西出口。加快推进“三旧”改造和棚户区、城中村改造，盘活存量建设用地资源，集约节约利用土地，提升用地水平。加大城市综合执法力度，铁腕整治违法用地违法建设。坚持建管并重，多管齐下，启动云山东江大桥、环状无障碍城市快速路建设。开展公共交通示范城市建设，优先发展公共交通。优化交通组织，采取“一个堵点一套方案”的精细化管理措施，努力缓解行车拥堵和停车难问题。

　　进一步发展壮大县镇经济。大力推进 “县区赶超计划”，提升县区综合实力。加强对县区产业发展的指导和扶持，加大财政对薄弱地区转移支付力度。实施大亚湾开发区对口帮扶惠东县、仲恺高新区对口帮扶龙门县，实现惠东县人均GDP超过全国平均水平、龙门县经济发展有长足进步。推动博罗、惠东、龙门三个县城扩容提质。强化规划引领，完成乡镇总体规划、中心镇近期建设用地控制性详细规划编制。启动市级专业镇建设计划，新培育认定市级专业镇10个。落实专项资金，扶持专业镇发展特色产业，力争建成4个以上总产值超100亿元的专业镇。大力推进名镇名村建设，全面增强专业镇产业、人口、资源集聚能力和承载能力。

　　进一步加强“三农”工作。不断加大强农惠农富农政策力度，健全“三农”投入稳定增长机制。深入开展粮食稳产增产行动，建成6个现代农业综合示范区和21.4万亩高标准基本农田，提高粮食安全保障水平。大力推动“菜篮子”基地建设，加快发展特色效益农业，确保全市特色农产品种植面积保持在200万亩以上。加快发展家庭农场和专业大户等新型现代农业经营主体，新培育市级以上农业龙头企业10家。加快农业科技创新，推行绿色生产，保障农产品质量安全。突出产业扶贫和智力扶贫，深入推进新一轮扶贫开发。完成25个省级贫困村通自来水工程。切实加大政策、资金扶持力度，努力改善革命老区生产生活条件，促进老区经济社会加快发展。

　　（七）积极加强生态建设，全面提升宜居环境质量。

　　坚持把生态建设放在更加突出位置，既要金山银山，又要绿水青山，努力走出一条具有惠州特色的生态文明建设新路子，争当珠三角城市群绿色生态示范市。

　　确保成功创建国家森林城市。全面推进新一轮绿化惠州大行动，加快森林碳汇、生态景观林带、森林进城围城、乡村绿化美化四大工程建设，提升林业生态建设水平。继续推进东江、西枝江水源涵养林和沿海防护林建设，构建森林生态安全体系，努力以更高质量成功创建国家森林城市。

　　全面加强环境保护和生态建设。落实“十二五”后半期节能行动计划和主要污染物总量减排行动计划，大力淘汰落后产能，确保完成省下达的节能减排降耗目标任务。深入实施“南粤水更清”行动计划，继续推进淡水河、潼湖流域污染综合整治，完成青年河小流域和水环境综合整治工程，启动望江沥、新开河综合整治工程。切实加强大气污染和扬尘污染防治，全力保持良好空气质量。推广应用新能源汽车。加快推进潼湖湿地公园、植物园规划建设。加强海洋生态保护，抓好海洋生态园、考洲洋海洋公园规划建设。在惠城中心区全面实施垃圾分类处理，推行县城垃圾分类试点，深入开展“美丽乡村·清洁先行”专项活动。扎实推进国家生态市创建，创建国家级生态镇10个、省级生态镇30个、省级生态县区2个。

　　建立健全生态保护机制。编制全市生态低碳规划，基本完成生态红线划定。实施生态公益林损失性补偿、市域内基本农田指标转移补偿，探索建立县（区）间横向生态补偿机制。严格执行环评制度，严把环保准入关。实施最严格的饮用水保护管理制度，用最严厉的法律手段确保东江、西枝江水质安全。以环大亚湾新区为试点，建立海陆统筹的生态系统保护修复机制，加快建设美丽港湾。

　　（八）全力抓好民生实事，加快发展社会各项事业。

　　坚持民生优先、发展惠民，市级新增财力75%以上、县级新增财力60%以上用于发展民生事业，推动民生质量不断提升。

　　扎实办好十件民生实事。今年全市投入77.99亿元，集中力量办好教育、卫生、文化、计生、社保、养老、助残、交通、环保、政务等十方面69项民生实事（具体项目见附件）。我们将切实落实责任，加强统筹协调，强化督查督办，确保全面完成各项任务。

　　提高基本公共服务均等化水平。坚持尽力而为、量力而行，扩大基本公共服务涵盖范围，提高保障标准。继续扩大市级基本公共服务均等化专项统筹资金规模，优化和扩展底线均等保障项目，提高基本公共服务保障能力。推进基本公共服务提供主体多元化、提供方式市场化、选择方式自主化。

　　推动教育事业优质均衡发展。启动“学区制和九年一贯对口招生”试点，抓好义务教育公办学校标准化建设，推进义务教育均衡发展示范项目。力争惠东、博罗、龙门通过省推进教育现代化先进县督导验收，提前一年创建成为省推进教育现代化先进市。建设惠州一中实验学校、惠州市实验中学附属学校等优质学校。申办惠州工程职业技术学院，支持惠州学院、经济职业技术学院发展。鼓励扶持社会力量办学。继续推行电子教育券。完善家庭经济困难学生帮扶机制。

　　进一步增强社会保障功能。落实更加积极就业政策，新增城镇就业人员7万人，转移农村富余劳动力1万人。继续推进社会养老保险扩面征缴工作，完成社会保障卡“一卡通”全覆盖。完善大病二次补偿机制，实现异地就医多向联网、即时结算。发展社会福利、慈善事业，完善养老、助残服务体系，配合省扶贫基金会推进省扶贫基地建设，启动市救助管理站迁建工作。制订五年规划，着力抓好农村泥砖房改造。继续做好城乡低保、“五保”供养、双拥和优抚安置、城乡特困户生活与医疗救助等工作。

　　扎实推进文明创建。广泛开展“共筑中国梦、当好建设者、同铸文明城”宣传实践活动，推进“好人之城”建设和志愿服务制度化，积极培育和践行社会主义核心价值观。完善文明创建常态化机制，推动文明创建向基层延伸，支持龙门争创全国文明县城。加大综合整治力度，扎实做好迎检准备工作，确保夺取全国文明城市“三连冠”。

　　加快建设文化惠州。推进文化场馆标准化建设，实现基层文化设施全覆盖，建成城市“十分钟文化圈”、农村“十里文化圈”。积极创建国家第二批基本公共文化服务体系示范项目。启动市工人文化宫建设。筹建未成年人综合素质教育基地。加快推进水东街改造工程，推进东坡祠等重要历史建筑重建以及宾兴馆、银岗古窑场保护开发，启动市美术馆、城市规划馆建设、东江纵队纪念馆迁建和葛洪博物馆筹建，力争成功申报国家历史文化名城。大力推进龙门农民画产业园、读者（惠州）数字出版基地、罗浮山影视产业基地等项目建设，积极发展新闻传媒、文化创意、数字出版、动漫设计、会展博览等文化产业。

　　全力创建平安惠州。大力推进平安建设十大工程项目建设，完善立体化、网格化治安防控体系，推动全警上路“大巡防”向全民参与“大群防”转变，重拳打击毒品犯罪和“两抢一盗”等多发性犯罪，扫除“黄赌毒”等社会丑恶现象，进一步巩固“中国最安全城市”建设成果。加强信访维稳和人民调解工作，扎实推进“社会矛盾化解年”各项工作，积极回应、及时解决群众合理诉求。全面落实安全生产责任，强化安全生产常态化监管、应急管理、宣传培训，预防和减少生产安全事故的发生。加强食品药品安全监管、产品质量管理和价格监督检查，打击走私贩私、制假售假、传销和变相传销，进一步规范市场经济秩序。

　　加快发展卫生计生等各项事业。深化医药卫生体制改革，加快推进公立医院改革。启动市中心人民医院总部建设，推进市第一人民医院扩建。完善基层医改运行新机制。推行平价医疗、平价药包。严格落实计生层级动态管理责任制，完善计生“惠一生”利益导向机制和“惠一家”优生健康工程。全力争创全国双拥模范城“五连冠”。启动社区体育公园建设，广泛开展全民健身运动，筹办好2014年中国龙舟公开赛暨第三届惠州国际龙舟邀请赛、2014年全国公路自行车冠军赛暨惠州市自行车公开赛、广东省第五届少数民族传统体育运动会等重大赛事活动。做好全国第三次经济普查。积极发展妇女儿童、青少年、红十字等事业，启动市流浪未成年人救助保护中心、妇女儿童庇护中心和市应急避灾中心建设。依法推进民族、宗教事务管理。继续做好国防动员、外事侨务、防震减灾、三防、消防、应急、台务、仲裁、方志、档案、气象、移民、科普、打私等工作，推动社会各项事业全面进步。

　　做好各项工作、完成预期目标，必须大力加强政府自身建设。我们将自觉接受人大及其常委会的法律监督、人民政协的民主监督和人民群众的舆论监督，认真落实人大及其常委会各项决议决定，健全重大事项向人大及其常委会报告和向政协通报制度，广泛听取民主党派、工商联、无党派人士和人民团体的意见，认真办好议案、提案、建议。坚持依法行政，深化政务公开，努力建设法治政府。深入贯彻中央八项规定，进一步落实国务院“约法三章”。扎实开展党的群众路线教育实践活动，切实解决“四风”突出问题。加强审计、监察工作，严肃财经纪律。严格落实党政机关厉行节约反对浪费条例，严控“三公”经费，最大限度压缩政府开支。深化廉政建设体制机制改革，完善惩治和预防腐败体系，努力建设廉洁高效、奋发有为的公务员队伍，努力打造为民务实清廉的人民政府。

　　各位代表，惠州已经步入全面深化改革的新时期，踏上“尽快进入珠三角第二梯队”的新征程。面对惠州发展的宏伟蓝图，面对父老乡亲的殷切期盼，我们必须勇于担当、真抓实干。让我们在省委、省政府和市委的坚强领导下，凝心聚力，奋力拼搏，全力确保今年各项目标任务圆满完成，不断开创经济社会发展新局面，为我市尽快进入珠三角第二梯队而努力奋斗！
